# Bone health in ambulatory male patients with chronic obstructive airway disease – A case control study from India

**DOI:** 10.1002/agm2.12239

**Published:** 2023-01-12

**Authors:** Mohammad Sadiq Jeeyavudeen, Samuel George Hansdek, Nihal Thomas, Thangakunam Balamugesh, Mahasampath Gowri, Thomas V. Paul

**Affiliations:** ^1^ Department of Endocrinology and Metabolism University Hospitals of Edinburgh Edinburgh UK; ^2^ Department of General Medicine Christian Medical College and Hospital Vellore India; ^3^ Department of Endocrinology, Diabetes, and Metabolism Christian Medical College and Hospital Vellore India; ^4^ Department of Pulmonary Medicine Christian Medical College and Hospital Vellore India; ^5^ Department of Biostatistics Christian Medical College and Hospital Vellore India

**Keywords:** COPD, osteopenia, osteoporosis, vitamin D deficiency

## Abstract

**Objective:**

Chronic obstructive airway disease (COPD) is characterized by airflow limitation due to airway and/or alveolar abnormalities with significant extra‐pulmonary manifestations. Bone health impairment is an extra‐pulmonary complication of COPD which is less well studied in India. Moreover, it can contribute to significant morbidity and mortality. Hence, we aim to estimate the prevalence of osteoporosis and metabolic parameters of adverse bone health in patients with COPD.

**Methods:**

In this case control study, male subjects aged 40–70 years with COPD attending the respiratory outpatient clinic in a tertiary care hospital were recruited over a period of 2 years and the control population were derived from the historical cohort who were apparently healthy with no obvious diseases. Metabolic parameters of bone health measured from fasting blood samples were calcium, albumin, alkaline phosphatase, phosphorous, parathormone, creatinine, 25‐hydroxy vitamin D, and testosterone. Bone mineral density (BMD) was estimated using DXA scan and the World Health Organization (WHO) criteria was used to categorize into osteoporosis, osteopenia, and normal BMD based on the T‐score at femoral neck, lumbar spine and distal forearm. Pulmonary function tests and 6 minute walk test were performed if they had not been done in the previous 3 months. The associations of COPD with osteoporosis were analyzed using linear regression analysis and effect size are presented as beta with 95% confidence interval.

**Results:**

Of the 67 participants with COPD enrolled in the study, osteoporosis was present in 61% (41/67) and osteopenia in an additional 33% (22/67) of the cases, which was higher when compared to the control population (osteoporosis 20% [50/252] and osteopenia 58% [146/252]). In regression modeling, there was a trend toward adverse bone health with advanced age, low body mass index, low forced expiratory volume in 1 second and testosterone deficiency in COPD.

**Conclusion:**

Individuals with COPD have a substantially higher prevalence of osteoporosis and osteopenia, up to almost twice that of the general population, with a significant number demonstrating at least one parameter of adverse metabolic bone health on assessment. Hence, bone health assessment should be a part of comprehensive COPD care to prevent adverse consequences due to poor bone health.

## Graphical abstract

Chronic obstructive airway disease (COPD) is a multimorbid disorder with two thirds affected have at least one extra‐pulmonary complication. Bone health in COPD is least studied in developing nations and, in our study, we have reported that osteoporosis is twice more common in COPD than in healthy individuals and with a significant number demonstrating at least one parameter of adverse metabolic bone health on assessment.

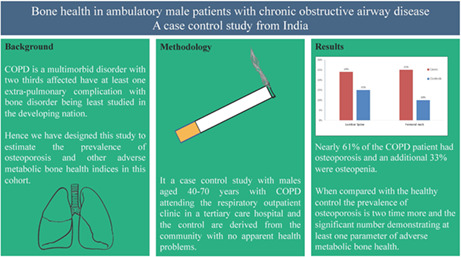

## 1 INTRODUCTION

Global Initiative for Chronic Obstructive Lung Disease (GOLD), defines chronic obstructive pulmonary disease (COPD) as a progressive disease characterized by persistent airflow limitation.^1^ COPD is a preventable and treatable disease; however, it contributes to significant morbidity in affected individuals due to its pulmonary and extra‐pulmonary effects. The burden of COPD is steadily increasing both in developed and developing countries. The recent World Health Organization (WHO) report estimates that around 328 million people around the world are living with moderate to severe COPD and more than 3 million deaths in 2005 were attributed to COPD or its systemic complications.^2^ This corresponds to 5% of deaths reported globally, although this number may be higher given that 90% of deaths occurred in developing countries where the reporting systems are suboptimal. COPD is the second leading cause of disease burden in India, contributing to 8.7% of the total deaths and 4.8% of the total disability adjusted life years (DALYs).^3^, ^4^, ^5^ Death due to COPD is higher in male patients, and people with longer disease duration, frequent exacerbations, and significant extrapulmonary complications.^6^

With advances in the treatment of COPD over the last 2 decades, people live longer, with more than two thirds affected by at least one extrapulmonary complication.^6^, ^7^ Cardiovascular comorbidity is one of the most feared extra pulmonary complications, characterized by increased incidence of systemic and pulmonary arterial hypertension, congestive cardiac failure, and arrhythmias.^8^ In a study by De Luise et al, there was a significant increase in the 30‐day mortality after a hip fracture in patients with COPD when compared with patients without COPD.^9^ This additional risk extends well beyond the immediate postoperative period with the mortality rate reaching nearly three folds even after a year. Hence, non‐communicable diseases, like osteoporosis, has emerged to significantly contribute to the disease morbidity and mortality. The increased risk of osteoporosis in patients with COPD has been attributed to the systemic nature of the disease and its treatment, which requires glucocorticoids, especially with those with frequent exacerbation.^10^

Major societal guidelines do not recommend COPD as risk factor for osteoporosis screening.^11^, ^12^ Fracture Risk Assessment (FRAX), one of the most popular assessment tools, does not include COPD as a risk factor in its assessment algorithm but has current smoking and glucocorticoid use as factors contributing to higher risk score.^13^ QFracture, another commonly used risk assessment tool, includes COPD as a risk factor for major osteoporotic fracture.^14^ Both these risk scores do not take into account factors like dose and repeated exposure to oral steroid and high dose inhaled glucocorticoids, which are commonly used for exacerbation in patients with uncontrolled COPD, and can independently predispose them to increased risk of fracture and added morbidity. There is also paucity of data on bone health in patients with COPD in developing countries like India. Hence, we have designed this study to estimate the prevalence of osteoporosis and other metabolic bone health indices in this cohort of patients.

## 2 SUBJECTS AND METHODS

This was a case control study conducted between September 1, 2012, and June 30, 2014. The study was approved by the institutional review board. The cases were consecutive male patients with COPD between 50 and 70 years of age attending the Respiratory Medicine outpatient services were screened, and those with known COPD, or newly diagnosed to have COPD as per the GOLD criteria, were enrolled into the study.^1^ Subjects of this age and gender were selected to homogenize the study population and to minimize the influence of hormonal changes affecting bone health seen in the extreme of ages, particularly in women. Subjects with hyperthyroidism, hyperparathyroidism, Cushing's syndrome or any other severe systemic illness, immobilization, and those who were already on calcium and vitamin D were excluded from the study. The control population was derived from the cluster random sampling of 242 individuals from the community who were apparently healthy without COPD and were of similar age and gender to the cases.^15^ They were also from the same region, and this was done to avoid the confounding effect of ethnicity influencing bone health. The prevalence of osteoporosis in the control population at any site was 20% (15% at the lumbar spine and 10% at the femoral neck), and further details of this study can be found elsewhere.^15^

Written informed consent was obtained from all subjects. Data were obtained regarding age, symptoms, exacerbation triggers of COPD, and the severity of the disease. A detailed medication history, including oral and inhaled glucocorticoid frequency, dose, and duration were documented along with the presence of pre‐existing comorbidities (eg, diabetes, hypertension, and dyslipidemia). The doses of inhaled glucocorticoids were calculated for the budesonide equivalent dose. Patients were then categorized into high dose and less than high dose based on the cumulative daily inhaled glucocorticoids dose. The high dose category patient received a cumulative dose of budesonide > 800 μg/day and the latter received less than 800 μg/day. The cumulative dose of oral glucocorticoids was calculated for the prednisolone equivalent dose. A validated semiquantitative food frequency questionnaire (FFQ) was used to calculate the dietary calcium intake by 24‐hour dietary recall method.^16^ Sunlight exposure was calculated from the duration for which the patient's body surface area was directly exposed to the sunlight such that when the shadow formed is smaller than the real image.^17^

All subjects underwent spirometry using the Jaeger spirometer and a 6‐minute walk test to make assessments as per the American Thoracic Society Guidelines.^18^ The GOLD criteria were used to categorize patients into the various disease stages.^1^ The body mass index, airflow obstruction, dyspnea, and exercise (BODE) index, which is a composite marker of disease severity that takes into consideration of the systemic nature of the disease, was calculated for all patients.^19^ The mortality risk according to the BODE index is as follows: a score greater than 7 is associated with a 30% 2‐year mortality, a score of 5–7 is associated with a 15% 2‐year mortality and < 5 is associated with 10% 2‐year mortality, respectively.^20^ Assessment of bone mineral density (BMD) was performed using the Hologic DXA Discovery QDR 4500 at lumbar spine, femoral neck, and distal forearm by the same technician. The reference standard consisted of healthy young White subjects used by the manufacturer's database with precision of 2% and the WHO criteria for osteoporosis based on T‐score were used to categorize the patients.^21^

Early morning fasting blood samples were collected in order to assess the following metabolic bone and other biochemical parameters: serum calcium (normal [N]: 8.3–10.4 mg/dL), phosphorus (N: 2.5–4.6 mg/dL), albumin (N: 3.5–5.0 g/dL), alkaline phosphatase (ALP; N: 40–125 U/L), creatinine (N: 0.5–1.4 mg/dL), 25‐hydroxyvitamin D_3_ (25[OH]D; N: 30–70 ng/mL), intact parathyroid hormone (iPTH; N: 8–50 pg/mL) and C‐reactive protein (CRP; N: < 6 mg/L), total testosterone (N: 300–1030 ng/dL), and cortisol (N: 7–25 μg/dL). The biochemical variables, such as calcium, phosphorus, creatinine, albumin, and ALP were measured in a fully automated computerized microanalyzer (Hitachi model 911; Boehringer Mannheim). The intra‐assay and inter‐assay coefficients of variation of the variables being studied from these machines were 1%–5%. Intact PTH, testosterone, and 25(OH)vitamin D were measured by a chemiluminescence immunoassay using an Immulite analyzer 2000. Vitamin D level was defined as sufficient for 25 (OH) D levels more than 30 ng/mL and deficient for levels < 20 ng/mL. CRP was estimated by immunonephelometry (BN ProSpec; Dade Behring) according to the manufacturer protocol using the CardioPhase highly sensitive CRP reagents. Hypogonadism was defined as 8 am total serum testosterone < 300 ng/dL.

## 3 SAMPLE SIZE CALCULATION AND STATISTICAL ANALYSIS

The sample size was calculated using prevalence data from a previously published study from India.^14^ A sample size of 64 subjects was required to study the prevalence of low bone density (osteoporosis and osteopenia) assuming a prevalence of 80% based on the previous Indian study using the equation 4 pq/d^2^ with a precision of 10%. The continuous variables were described using means and standard deviations or median and interquartile range (IQR) depending on normality. All categorical variables were summarized by using frequencies and percentages. Association for continuous variables with low bone density was done using Independent *t* test and for categorical associations chi‐square test was used. The T‐scores of each region were considered as continuous outcome as the larger percent of the cohort has either osteopenia or osteoporosis. Linear regression model was used to determine significant predictors. Univariate model was used to define the individual effect of each predictor. Multivariate model was constructed adjusting for variables with entry criteria of *P* value < 0.20. The effect sizes were presented with beta (and 95% confidence interval [CI]). For all analyses, the significance level was determined for *P* < 0.05. The results of this study were compared with a historical cohort of previously published subjects from the same ethnicity without COPD.^15^ All statistical analyses were done using STATA/IC version 16.0.

## 4 RESULTS

This study included 67 male subjects diagnosed with COPD based on the GOLD criteria. The mean (±SD) age group of the study population was 60 (±6) years, and the mean duration of COPD was 48 months (Table 1).

**TABLE 1:** Baseline characteristics of the study subjects

	Overall (n = 67)	Normal (n = 6)	Osteopenia (n = 33)	Osteoporosis (n = 28)	*P* value^d^
Age (y)^a^	60.2 ± 6.9	59.5 ± 6.8	59.2 ± 7.3	61.6 ± 6.4	0.176
Current smokers^c^	7 (10)	0 (0)	2 (6.1)	5 (17.9)	0.093
No. of pack years^b^	30 (20, 46.5)	30 (28, 40)	24 (15, 44.5)	36 (25, 50)	0.176
Duration of COPD in months^b^	48 (24, 72)	18 (12, 39)	60 (36, 84)	54 (24, 72)	0.673
6 MWD (meters)^a^	348 ± 92.1	318.9 ± 84.3	370.2 ± 97.1	328 (84)	0.134
FEV1^a^	42.2 ± 18.6	51.9 ± 21.8	44.3 ± 19.6	37.5 ± 16	0.085
FVC^a^	61.3 ± 17.2	71.8 ± 14.9	61 ± 18.8	59.5 ± 15.4	0.464
FEV1/FVC^a^	67.6 ± 17.5	70.3 ± 20.9	72.2 ± 18.8	61.7 ± 13.4	0.017
Oral steroid dose^b^	0 (0, 0)	0 (0, 0)	0 (0, 0)	0 (0, 20)	0.287
Oral steroid duration in the last 1 y^b^	0 (0, 0)	0 (0, 0)	0 (0, 0)	0 (0, 5)	0.282
Dietary calcium intake^b^	1156.3 ± 264.2	1048.3 ± 231.4	1157.9 ± 291.6	1177.5 ± 238.5	0.581

Abbreviations: 6 MWD, 6 minute walking distance; COPD, chronic obstructive pulmonary disease; FEV1, forced expiratory volume in the first second; FVC, forced vital capacity; IQR, interquartile range.

^a^ Summarized using mean ± SD.

^b^ Summarized using median (IQR).

^c^ Presented as n (%).

^d^ The *P* values are presented comparing osteoporosis as one group and osteopenia and normal clubbed together.

The majority of the patients were distributed equally in stages II, III, and IV, there was only one patient with stage I disease. The frequency of patients in three BODE categories‐ < 5, 5–7 and more than 7 were 8, 7 and 52 patients, respectively. Nine of the study participants received high dose inhaled glucocorticoids of which one had osteoporosis and the rest had osteopenia. Seven patients received oral glucocorticoids in the last 2 years. As expected, these patients were in stages III and IV disease category and had a high BODE index score. The prevalence of vitamin D deficiency was 52% (N: 35/67). Biochemical hypogonadism was seen in 31% (N: 21/67). Duration of sunlight exposure was equal in all the groups.

**FIGURE 1:**
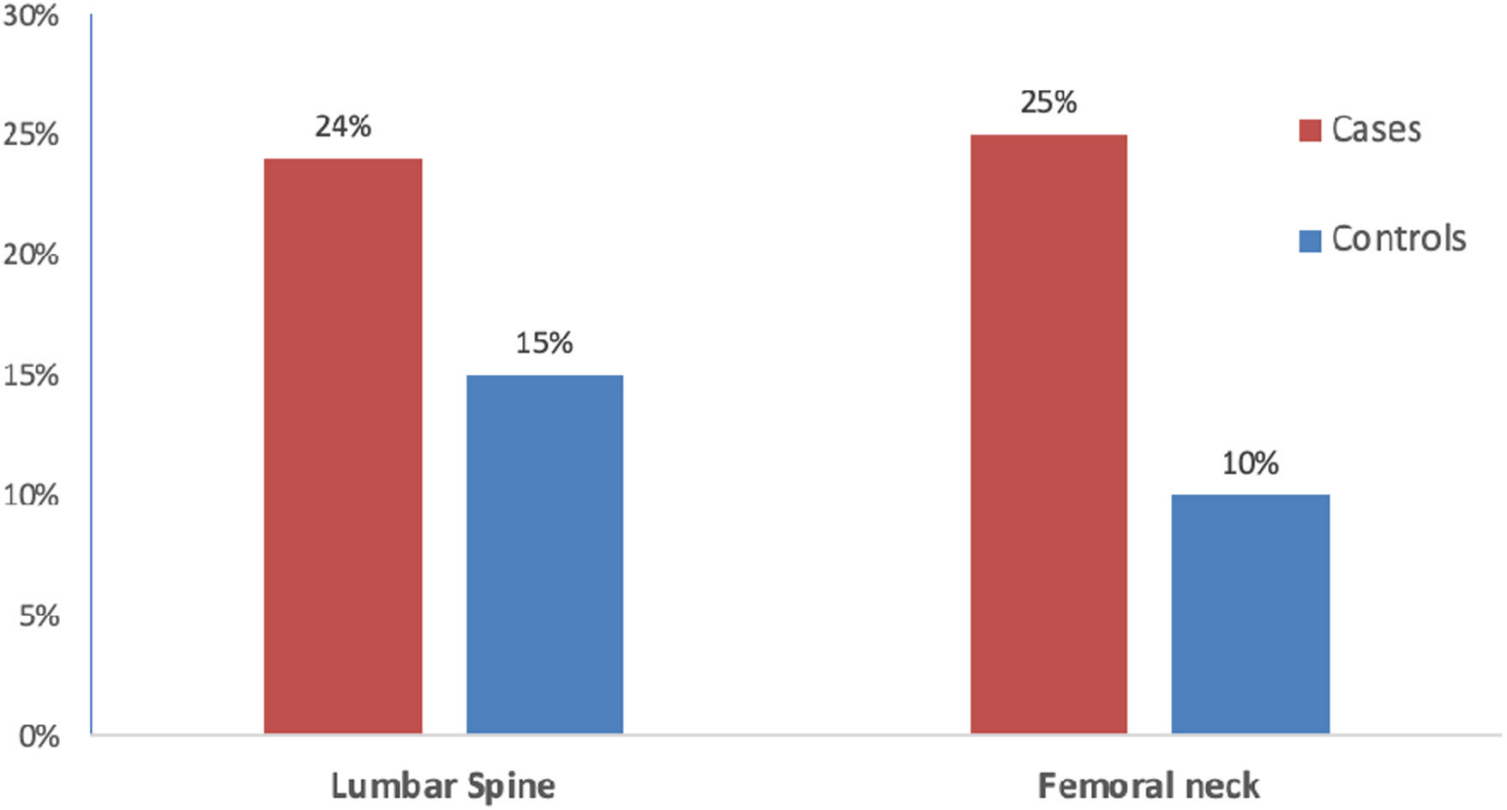
Prevalence of osteoporosis between cases and controls across different sites.

The prevalence of osteoporosis at any one site in this study was found to be 61% (41/67). The prevalence of osteoporosis at the lumbar spine and femoral neck were almost equal with 24% (16/67) at the lumbar spine and 25% (17/67) at the femoral neck. The prevalence of osteopenia at the lumbar spine and femoral neck was found to be 47% (31/67) and 53% (36/67), respectively. There was an increased prevalence of osteoporosis of 33% (22/67) and osteopenia 33% (22/67) at the distal forearm compared to the other sites (Figure 1).

In the univariate regression model, at least one site of lower T‐score for osteoporosis in male patients with COPD were significantly associated with age, body mass index (BMI), smoking status, forced expiratory volume in 1 second (FEV1) and FEV1/FVC (Table 2). BMI remained significantly associated with lower T‐score even in the multivariate analysis (Table 3).

**TABLE 2:** Univariate associations of T‐scores at each site with clinical and demographical variables

Variables	Lumbar spine	Femoral neck	Distal forearm
Age, y	0.033 (−0.016, 0.081)	−0.022 (−0.056, 0.011)	−0.066 (−0.121, −0.011)*
BMI	0.096 (0.034, 0.158)**	0.084 (0.042, 0.125)**	0.124 (0.053, 0.196)**
Current smoker	−0.022 (−1.112, 1.068)	−0.516 (−1.268, 0.236)	−1.294 (−2.533, −0.055)*
FEV1	0.021 (0.003, 0.038)*	0.012 (0.000, 0.025)	0.007 (−0.014, 0.028)
FEV1/FVC	0.024 (0.005, 0.042)*	0.012 (−0.001, 0.026)	0.014 (−0.008, 0.037)
GOLD stage	−0.318 (−0.735, 0.100)	−0.121 (−0.420, 0.179)	0.059 (−0.447, 0.566)
BODE index (> 7)	−0.220 (−0.884, 0.444)	−0.128 (−0.593, 0.337)	0.240 (−0.540, 1.021)
25(OH) vitamin D	0.079 (−0.322, 0.480)	−0.020 (−0.301, 0.261)	−0.227 (−0.695, 0.241)
Serum iPTH	−0.107 (−0.783, 0.568)	0.015 (−0.458, 0.487)	0.223 (−0.568, 1.015)
Testosterone level (≥ 300 ng/dL)	0.244 (−0.472, 0.960)	0.292 (−0.206, 0.789)	−0.067 (−0.911, 0.777)

Abbreviations: BMI, body mass index; BODE, body‐mass index, airflow obstruction, dyspnea, and exercise; CI, confidence interval; FEV1, forced expiratory volume in the first second; FVC, forced vital capacity; GOLD, Global Initiative for Chronic Obstructive Lung Disease; iPTH, intact parathyroid hormone.

*Note*: Each cell represents a single regression equation coefficient (95% CI); ***P < 0.001; **P < 0.01; *P < 0.05.

**TABLE 3:** Multivariate associations of segmental t‐scores with clinical and demographic variables

Variables	Lumbar spine	Femoral neck	Distal forearm
Age, y	0.041 (−0.007, 0.090)	−0.016 (−0.049, 0.016)	−0.056 (−0.111, −0.001)
BMI	0.105 (0.031, 0.179)**	0.064 (0.014, 0.113)*	0.064 (−0.020, 0.148)
Current smokers	0.351 (−0.738, 1.440)	−0.292 (−1.028, 0.445)	−1.002 (−2.244, 0.240)
FEV1	0.014 (−0.017, 0.044)	0.017 (−0.003, 0.038)	0.026 (−0.008, 0.061)
BODE index (> 7)	0.105 (−1.062, 1.271)	0.435 (−0.354, 1.223)	0.975 (−0.356, 2.306)
Testosterone level (≥ 300 ng/dL)	0.358 (−0.330, 1.046)	0.413 (−0.052, 0.878)	0.198 (−0.587, 0.982)

Abbreviations: BMI, body mass index; BODE, body‐mass index, airflow obstruction, dyspnea, and exercise; CI, confidence interval; FEV1, forced expiratoryvolume in the first second.

*Note*: Each row represents a single regression equation coefficient (95% CI); ***P < 0.001; **P < 0.01; *P < 0.05.

The mean BMD in the present study was compared with age and gender‐matched controls without COPD or other chronic disease affecting bone health (Table 4).^15^ The mean BMD at the femoral neck for patients with COPD (0.692 kg/m^2^) was significantly lower when compared with healthy subjects of similar age group, ethnicity, and gender (0.761 kg/m^2^, *P* < 0.001). A similar finding was also found in the lumbar spine region (mean BMD patient with COPD: 0.906 kg/m^2^ vs. normal subject 0.943 kg/m^2^, *P* = 0.024).

**TABLE 4:** Biochemical parameters and BMD of the study subjects compared with the historical cohort

Parameters	COPD (n = 67) Mean (SD)	Non‐COPD^15^ (n = 252) Mean (SD)	Unpaired *t* test *P* value
Serum calcium (mg/dL)	9.32 (0.56)	8.82 (0.43)	< 0.001
Serum PO_4_ (mg/dL)	3.65 (0.75)	3.9 (0.5)	0.001
Serum iPTH (pg/mL)	57.11 (28.59)	44.5 (25.6)	< 0.001
Serum alkaline PO_4_ (U/L)	83.84 (28.42)	73.5 (21.4)	0.001
Serum 25 OH vitamin D (ng/mL)	25.25 (16.50)	20.4 (8.3)	< 0.001
Serum testosterone (ng/dL)	381.15 (173.71)	620 (124)	< 0.001
ESR (mm/h)	17.37 (12.93)	–	
CRP (mg/L)	11.04 (13.89)	–	
Bone mineral density			
Femoral neck (g/cm^2^)	0.692 (0.130)	0.761 (0.124)	< 0.001
Lumbar spine (g/cm^2^)	0.906 (0.145)	0.943 (0.111)	0.024
Distal forearm (g/cm^2^)	0.588 (0.089)	–	

Abbreviations: BMI, body mass index; COPD, chronic obstructive pulmonary disease; ESR, erythrocyte segmentation rate; iPTH, intact parathyroid hormone.

## 5 DISCUSSION

In the current study, the prevalence of osteoporosis in men with COPD was 61%, and hypovitaminosis D was seen in 52% of the study subjects. These results along with the previously published data confirms that people with COPD have weaker bone mass, and prevalence of osteoporosis is nearly doubled when compared with healthy men in the same community (Table 4).^15^, ^22^, ^23^, ^24^ The osteoporosis prevalence from our study matches data from two other previously published reports from India. The first was published by Bhattacharya et al who measured BMD using calcaneal ultrasound.^22^ In the second study by Hattiholi et al, the prevalence of osteoporosis and osteopenia were 66.7% and 19.6%, respectively.^23^ However, other parameters relating to adverse bone health were not reported in both these studies. The prevalence of osteoporosis reported in these Indian studies were more when compared with Western studies.^25^, ^26^ In the multicentric TOwards A Revolution of COPD Health Study (TORCH trial), the prevalence of osteoporosis and osteopenia were 18% and 41%, respectively.^27^ The reason for an increased prevalence of osteoporosis in our study and other studies reported from India may be due to an increased community prevalence of osteoporosis and vitamin D deficiency, an advanced stage of the disease, and a higher dose of glucocorticoids used for treatment.^28^

The increased risk for osteoporosis in patients with COPD is due to the systemic nature of the disease, glucocorticoid intake, change in body composition and weight, decreased activity, reduced exercise reserve, and reduced sunlight exposure due to dyspnea associated with mobility during advanced stages of the disease. What causes this systemic dysfunction is not clearly understood, but there are some hypotheses that are postulated and tested. The two important ones are a systemic spillover theory and a compartment model. In the systemic spillover hypothesis, it is assumed that there is a spillover of the cytokines and inflammatory mediators due to chronic inflammation in the lungs into the systemic circulation.^29^, ^30^ The compartment model states that there are two or more compartments where the disease process is ongoing simultaneously.^31^, ^32^ The distant organ or systems affected, as mentioned earlier, were the cardiovascular system, adipose tissue, and bone, and the primary organs are the lungs.

The mean BMI of our study population was 23 kg/m^2^ (2 SD ± 5.06). BMI in our study population is similar to that seen in the other two studies reported from India as compared to the Western study population who have a much higher BMI.^22^, ^23^ In our study, BMI was positively correlated with the BMD. Mechanical bone loading increases the bone strength and remodeling but it also ultimately depends on the fat free mass that contributes to this increased effect.^33^ Fat free mass in patients with COPD has been reported to be low and this depends on the severity of disease category with a decrease of 20% in a clinically stable patient with COPD to 41% in severe cases those requiring pulmonary rehabilitation when compared to the age and gender matched general population.^34^ Leptin, an adipocyte derived hormone has a biphasic effect on bone modeling and re‐modeling. At low concentration, it promotes proliferation and differentiation of osteoblasts but at high concentration it inhibits the bone formation both through central and peripheral effects.^35^ Moreover, this effect of leptin is more pronounced in obese women with COPD, who have high circulating leptin levels.^36^ Hence, body weight and BMI have a complicated relationship with bone health.

The other parameters that were significant in the regression modeling were testosterone deficiency and FEV1 level. It is well‐established that testosterone has positive effects on bone formation by its direct action and indirect action through aromatization to estrogen.^37^ Testosterone exerts its direct effects by binding to androgen receptors expressed on the pre‐osteoblast and helps its maturation whereas estrogen influences bone formation and inhibits resorption through its action on the estrogen receptor.^21^ FEV1 had a positive effect on the bone health and is likely related to the systemic state of the patient, as a higher FEV1 indicates better lung function. Hence, this will make the individual mobilize better for proper bone loading, sunlight exposure, and lower steroid requirements for disease control. The inflammatory markers, erythrocyte segmentation rate (ESR) and CRP were elevated in our study population. Suppression of bone formation and an increase in osteoclastogenesis in chronic inflammatory disease has been shown to induce proteins, such as Dickopf 1 and sclerostin.^38^ By inhibition of the Wnt pathway, these proteins along with several other cytokines, such as IL‐15, interferon gamma, IL‐17 MCP‐4 (monocyte chemoattractant protein), and TNF‐α blunt the bone formation there by leading to osteoporosis and its sequelae.^39^, ^40^

Regular use of oral glucocorticoids significantly increases the risk of osteoporosis.^41^ This is due to the uncoupling of bone formation as well as due to the direct toxic effect of steroids on the osteoblast. High dose inhaled glucocorticoids are known to have systemic effects with adverse bone effects and dose‐related adrenal suppression.^42^ Our study had only nine participants (14% percent) on high dose inhaled glucocorticoid and this did not achieve statistical significance with adverse bone health, potentially due to the reduced sample size. But this finding is similar to TORCH trial, which did not show an increase in bone loss in people taking inhaled glucocorticoids when compared with those on placebo.^27^

Although the study population resides in and around Vellore (Vellore, 12 degrees55′N, longitude 79 degrees11′E) where there is abundant sunlight throughout the year, only 13% had adequate exposure to sunshine. Sunlight is an abundant source for vitamin D, which in turn is an intermediate factor contributing to the bone health.^43^ Exposure to the sunlight should be at the time when the vitamin D synthesis is at its peak, and this usually happen at early noon when the ultraviolet B component of the sunlight is at its maximum. The surrogate marker for this in practical sense would be when the length of the shadow formed is less than the individual's height and the recommended duration of exposure is for at least 30 minutes.^28^ Because of restriction to outdoor activity, due to dyspnea, and in the late stages due to the requirement of oxygen therapy, this can be limited in patients with COPD. The dressing pattern among Indian men exposes only face and feet to sunlight when involved in outdoor activities. Hence, only 23% of our study population had sufficient 25(OH)D levels, which is less than community prevalence in a healthy individual. To our knowledge, we do not know any other study from India which has reported the prevalence of vitamin D deficiency in patients with COPD. Comparing our prevalence data with Western studies would be inappropriate, as the vitamin D synthesis due to sunlight exposure depends on the solar zenith angle, minimal erythema dose, duration of sunlight exposure, and dressing pattern.^44^, ^45^

The limitation of our study is the small sample size which precludes the possibility of making comparison across different stages of COPD. However, this is the first study from India, to our knowledge, to assess other parameters other than BMD to examine bone health in a male patient with COPD. It may be prudent to conduct similar studies in groups of premenopausal and postmenopausal women with COPD on a separate basis to understand the profile of their bone health.

## 6 CONCLUSION

Osteoporosis and an abnormal bone health profile is highly prevalent among patients with COPD. Differences in the patient characteristics and diagnostic tools account for the varied prevalence across studies, in any case, it is much higher than the general population. Higher prevalence of osteoporosis in the past was solely attributed to the increased glucocorticoid exposure but parameters for adverse bone health were seen even in steroid naive patients suggestive of a more complex underlying mechanism. Osteoporosis and osteoporotic fracture related morbidity and mortality will add to the already existing disease burden in those affected by COPD. But these can be prevented with proper screening and intervention, including lifestyle changes (increasing calcium intake in the diet and adequate sunlight exposure), vitamin D, calcium supplementation, and bisphosphonates when needed. This should be included in the comprehensive COPD care plan and modified to suit each individual patients’ needs.

## AUTHOR CONTRIBUTIONS

*Research and study design*: Jeeyavudeen, Hansdek, Thomas, Balamugesh, Gowri, and Paul. *Data collection*: Jeeyavudeen, Hansdek, Gowri, and Paul. *Data analysis*: Balamugesh, Gowri, and Paul. *Interpretation and conclusion*: Jeeyavudeen, Hansdek, Thomas, and Paul. *Preparation of manuscript*: Jeeyavudeen, Hansdek, and Paul. *Review of manuscript*: Jeeyavudeen, Hansdek, Thomas, Balamugesh, Gowri, and Paul. *Critical revision*: Jeeyavudeen, Hansdek, and Paul. Guarantors for the study: Jeeyavudeen.

## FUNDING INFORMATION

The protocol was approved by the institutional review board (IRB) of Christian Medical College, Vellore, and the funding was provided by the FLUID grant of the IRB. There was no involvement of the funding source in study design, in the collection, analysis, and interpretation of data, in the writing of the report, and in the decision to submit the paper for publication.

## CONFLICT OF INTEREST

The authors report no conflicts of interest for this study.

## ETHICAL APPROVAL

This study was approved by Office of Research, Institutional Review Board, Christian Medical College, Vellore, India IRB Min No: 7996 [Dated] February 12, 2013.
